# Translating the Brazilian Dietary Guidelines into clinical practice: innovative strategies for healthcare professionals

**DOI:** 10.20945/2359-4292-2024-0142

**Published:** 2025-01-27

**Authors:** Vanessa Del Castillo Silva Couto, Maria Laura da Costa Louzada, Patrícia Constante Jaime

**Affiliations:** 1 Núcleo de Pesquisas epidemiológicas em Nutrição e Saúde (NUPENS), Universidade de São Paulo, São Paulo, SP, Brasil; 2 Comissão Especial de Meio Ambiente, Sustentabilidade e Cultura Alimentar da Associação Brasileira para o Estudo da Obesidade (Abeso), São Paulo, SP, Brasil

**Keywords:** Diet, practice guideline, delivery of health care, health promotion, ultra-processed foods

## Abstract

TheBrazilian Dietary Guidelines provide crucial recommendations for a healthy diet, aiming at promoting health and preventing non-communicable chronic diseases. The core principle is the preference for natural or minimally processed foods and freshly prepared dishes over ultra-processed foods. Despite their growing recognition, healthcare professionals struggle to integrate these guidelines into clinical practice. This article aims to present two innovative strategies for incorporating the Brazilian Dietary Guidelines into healthcare. The Protocols based on the Brazilian Dietary Guidelines for Individual Dietary Advice are standardized clinical tools to support healthcare professionals (nutritionists or not) in giving nutritional advice during individual appointments to various life stages. The Protocols operationalize the assessment of individuals’ dietary patterns using the Food Consumption Markers Questionnaire and support the delivery of personalized and priority recommendations through a stepwise flowchart. Conversely, Brazilian Dietary Guidelines-based Meal Plans consist of personalized dietary prescriptions comprising structured daily menus that, unlike conventional plans primarily focusing on nutrient goals, prioritize overall eating patterns guided by the Brazilian Dietary Guidelines. The proposal encourages, in the first place, the selection of a variety of culinary preparations based on natural or minimally processed foods, emphasizing tasteful, accessible, and culturally appropriate choices as the initial step. In a second step, these plans can be customized to individual energy requirements, and adjustments made based on strategic nutrient needs. This article aims to support the enhancement of healthcare professionals’ skills in promoting healthy eating practices, thereby contributing to improved health and a reduced disease burden among the Brazilian population.

## INTRODUCTION

The Brazilian Dietary Guidelines provide official recommendations for a healthy diet for the Brazilian population (1). They are part of a set of actions for health promotion and prevention of non-communicable chronic diseases (NCDs). The Guidelines reflect secular changes in nutritional conditions of the Brazilian population and, grounded in the most robust scientific evidence, introduce a new paradigm that acknowledges the importance of considering the characteristics of industrial food processing in the diet-health relationship (2). Additionally, the Guidelines highlight how to combine foods into meals based on the traditional Brazilian diet, emphasize the importance of commensality and the environment that surrounds these meals, and provide guidance on strategies to overcome barriers to healthy eating (2).

Despite the recognition of the Dietary Guidelines’ importance as a guiding tool for policies and nutrition-related actions within the health system (3,4), and the understanding that promoting healthy eating should involve the multidisciplinary team (5), a significant challenge persists for healthcare professionals in incorporating its recommendations into the routine of clinical care throughout the life course (6-11). This challenge is extended to nutritionists or dietitians (considered as synonyms) who grapple with translating information from the Guidelines into dietary prescriptions (10-13). Considering that an unhealthy diet is currently one of the leading risk factors for the global burden of disease, addressing said challenge is imperative.

Previous studies demonstrated that developing implementation strategies is an important step in assisting professionals to overcome barriers in incorporating the recommendations into practice (9-11). So, this article aims to describe two innovative strategies on how the Brazilian Dietary Guidelines can be incorporated into the clinical practice of healthcare professionals, both nutritionists and non-nutritionists.

The initial section will provide an overview of the recommendations of the Brazilian Guidelines. Subsequently, the article will address the fundamental principles of dietary advice, exploring the incorporation of the Guidelines into clinical practice through two strategies: the Protocols based on the Brazilian Dietary Guidelines for Individual Dietary Advice and the Dietary Guidelines-based Meal Plans. The application of these tools will be illustrated by a clinical case.

## PREFER NATURAL OR MINIMALLY PROCESSED FOODS OVER ULTRA-PROCESSED FOODS: THE SCIENTIFIC EVIDENCE BEHIND THE BRAZILIAN GUIDELINES

The Brazilian Guidelines categorize foods according to the characteristics of the industrial processing. There are four groups: natural or minimally processed foods, culinary ingredients, processed foods, and ultra-processed foods (14).

Ultra-processed foods are industrial formulations typically developed from food derivatives or laboratory-synthesized substances. They are made from ingredients such as sugars, refined starches, isolated proteins, and remnants of intensively reared animals. Examples include cookies, sausages, sodas, dairy drinks, and ready-to-consume dishes.

The ingredients and processes involved in creating ultra-processed foods result in ready-to-consume products that are hyperpalatable, increase energy intake and easily replace other food groups (15,16). Across thirteen countries, the consumption of ultra-processed foods was inversely associated with the consumption of grains, legumes, fruits, and vegetables (15), and reduced dietary diversity (17,18). Their consumption is consistently associated with the deterioration of the nutritional dietary quality. The consumption of these foods is directly associated with unhealthy fats and free sugar intake, and inversely associated with fiber, and many micronutrients (15). A study with Brazilian adults also demonstrated an inverse association between the consumption of these foods and the intake of bioactive compounds relevant to cardiovascular health, such as flavonoids and phenolic acids (19). Lastly, their consumption is associated with biochemical markers of endocrine disruptors, including compounds formed during processing (acrylamide) or released from packaging materials (bisphenol A) (20,21).

A recent umbrella review presented the negative impact of these foods on health. The studies demonstrate that high consumption of ultra-processed foods is associated with a higher risk of more than 30 chronic diseases, including obesity, diabetes, cardiovascular diseases, inflammatory bowel disease, and depression (22-24). Mechanistic studies have explicited that such associations are explained by combinations of many harmful features of ultra-processed foods that go beyond nutrients (25). In Brazil, household acquisition surveys showed that the dietary share of ultra-processed foods increased from 14.3% in 2002/2003 to 19.4%, in 2017/2018 (26), which was responsible for almost 30% of the increase in obesity prevalence in the same period (27).

Additional evidence comes from a trial comparing the effects of providing an ultra-processed diet (~80% ultra-processed foods) and an unprocessed diet (without ultra-processed foods). Participants could eat *ad libitum*, but the menus were identical in terms of calories and several nutrients. After two weeks, participants gained 0.9 kg in weight when consuming the ultra-processed diet and lost 0.9 kg in weight when consuming the unprocessed diet (28).

Corroborating this evidence, the Brazilian Dietary Guidelines establish their golden rule: “prefer natural or minimally processed foods and their culinary preparations over ultra-processed foods”. The Ten Steps to Healthy Diets are:

1. Make natural or minimally processed foods the basis of your diet.
2. Use oils, fats, salt, and sugar in small amounts when seasoning and cooking natural or minimally processed foods and to create culinary preparations.
3. Limit consumption of processed foods.
4. Avoid consumption of ultra-processed foods.
5. Eat regularly and carefully in appropriate environments and, whenever possible, in company.
6. Shop in places that offer a variety of natural or minimally processed foods.
7. Develop, exercise and share cooking skills.
8. Plan your time to make food and eating important in your life.
9. Out of home, prefer places that serve freshly made meals.
10. Be wary of food advertising and marketing.

## INNOVATIVE STRATEGIES TO TRANSLATE THE BRAZILIAN DIETARY GUIDELINES INTO CLINICAL PRACTICE

Incorporating the Dietary Guidelines into clinical practice presupposes understanding that dietary advice must take into account the social determinants of health. It shall be permeated by sensitivity and awareness of people’s life projects and how their diets are part of it. It should be based on the assessment of individuals’ eating habits and consider sociodemographic conditions, household, educational and workplace environments, functional capacity, as well as preferences, habit, and culture (29,30).

The evaluation of eating habits can be carried out with the Food Consumption Markers Questionnaire (31). This short questionnaire is part of the surveillance system of the Brazilian Health System and is routinely administered by primary care professionals, providing support for clinical practice (32). This questionnaire is capable of assessing the adherence to the main recommendations of the Brazilian Dietary Guidelines and can predict diet quality, considering the consumption of ultra-processed foods and key nutrients for NCDs (saturated fat, trans fat, added sugar, sodium, potassium, and fiber), as well as dietary diversity (33).

The questionnaire for individuals aged ≥2 years has 9 yes/no questions evaluating the consumption, on the previous day, of beans, fruits, vegetables, and a set of ultra-processed foods, as well as the habit of having meals while using screens, and the number of meals consumed throughout the day.

It is important to emphasize that additional questions will be necessary for the healthcare professional to provide specific dietary advice and to devise personalized strategies to overcome each person’s obstacles. For example, in the case of a person consuming sugar-sweetened beverages, the professional may ask additional questions to understand which beverage has been consumed, whether the consumption is habitual or occasional, at what time of day, and what other similar foods the person enjoys.

The choice of the best strategy to provide dietary advice will depend on various factors, ranging from the knowledge and self-efficacy of the healthcare professional to factors like time, willingness, complexity of clinical conditions, and the evaluation of the priorities of the individuals. Shared decisions between individuals and healthcare professionals, promoting self-care and autonomy, can lead to more enduring interventions (30,34).

The content of the Brazilian Dietary Guidelines, by moving away from a nutrient-centric perspective and considering a new way of categorizing foods and more holistic recommendations that address eating patterns, emerges as a tool that better alignes with the technical knowledge and practices of the multidisciplinary team, as well as with habits culturally recognized by the population. Therefore, it may support the implementation of more effective dietary advice (35,36).

A clinical case will provide practical examples of each stage in the implementation of dietary advice using the approaches proposed in this article: the Protocols Based on the Brazilian Dietary Guidelines for Individual Dietary Advice (hereafter called Protocols) and the Dietary Guidelines-based Meal Plans. In **Box 1**, we introduce Theresa (fictitious name), an elderly woman attended by a nurse at a primary care service, who conducts the assessment of her clinical conditions and her eating habits (part 1) and refers her to the family doctor (part 2) and the nutritionist (part 3).

**Figure:**
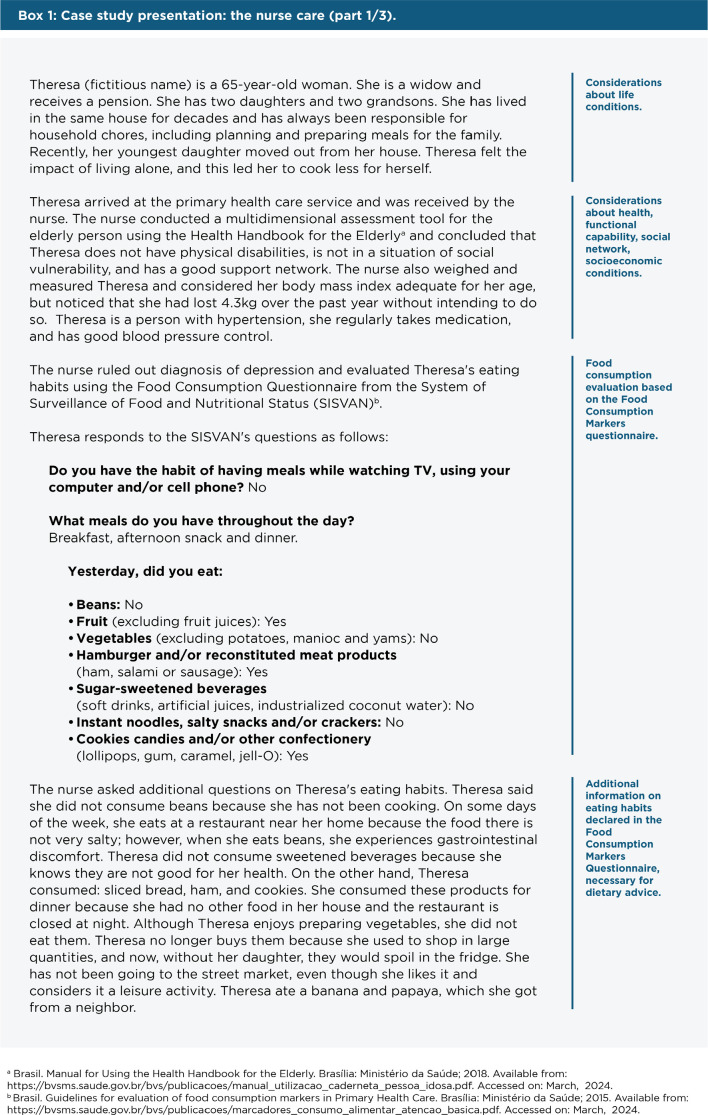

### Protocols based on the Brazilian Dietary Guidelines for Individual Dietary Advice

The Protocols are pioneer clinical standardized protocols developed to support healthcare professionals in giving nutritional advice during individual appointments (35). They were developed between 2020 and 2022 by the Center for Epidemiological Research in Nutrition and Health from the University of São Paulo (NUPENS/USP) and the Ministry of Health. The protocols are organized into 5 fascicles, each one providing dietary advice for a specific life stage: adults, the elderly, adolescents, children aged 2 to 10 years, and pregnant people (31-38). Each fascicle of the Protocols was validated by expert and health professionals panels, which reinforced that these tools are clear, easily understandable, and applicable, capable of supporting the provision of dietary advice (37-41). The methodological framework for the development and validation, as well as the adaptation for each life stage, are described in previous studies (35,42,43). The Protocols recognize that effective dietary advice requires consideration of the potential obstacles for each individual in improving dietary practices. This involves considering factors such as the individual’s workplace environment and functional capabilities. As such, they do not feature generalized messages applicable to everyone but rather include specific messages and distinct proposals of strategies to promote healthy dietary practices, which should be chosen by the healthcare professional according to each case.

**Figure 1:**
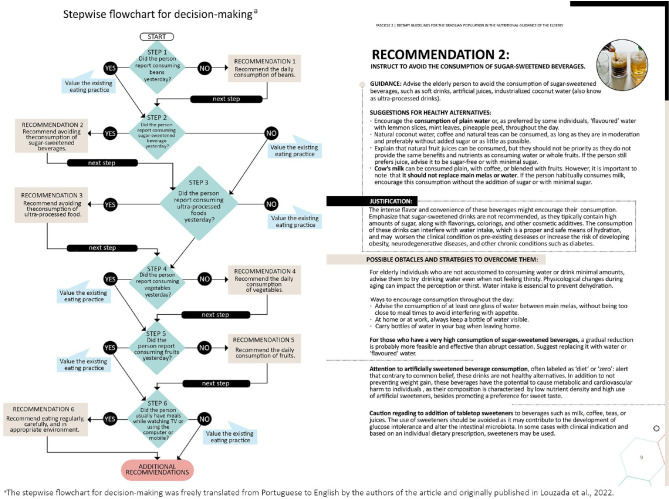
Reproduction of the Protocols' stepwise flowchart for decision-making and one set of recommendations

The Protocols follow 3 steps, briefly explained below:

1. Assessment of individuals’ food consumption based on the Food Consumption Markers Questionnaire (31).
2. Prioritization of recommendations based on the stepwise flowchart for decision-making: A stepwise flowchart supports the healthcare professional in organizing dietary advice and choosing the priority recommendations (**Figure 1**) based on the answers provided in the Food Consumption Markers Questionnaire. The flowchart prioritizes the golden rule of the Dietary Guidelines: “Always prefer natural or minimally processed foods and freshly made dishes and meals to ultra-processed foods”. Thus, it begins evaluating the consumption of beans, understanding it as a marker of high-quality natural food, typical of the Brazilian food culture, and the consumption of full meals that include other natural or minimally processed foods, such as rice. Secondly, it addresses the consumption of ultra-processed foods and describes various strategies to avoid their consumption. To improve and diversify the diet, it offers recommendations for the consumption of fruits and vegetables. Lastly, it offers recommendations on healthy eating modes. Additional recommendations provide information according to their life stage.
3. Implementation of dietary advice: In the last step, the healthcare professional is directed to the recommendation sections, following each stage of the stepwise flowchart for decision-making (**Figure 1**). Within each recommendation, there is a range of information that can be utilized by the healthcare professional, according to the life conditions and obstacles presented by each individual. There is no determination regarding the minimum or maximum number of recommendations that can be made during the appointment; therefore, healthcare professionals can be flexible to determine how many recommendations can be made based on available time and mutual agreement with the individual, and continuing the approach in the upcoming appointments.

The Protocols have the potential to bring about a paradigm shift in clinical practice by combining, in a single tool, a concise dietary practices assessment instrument, the most up-to-date healthy eating framework, and a delivery technique that promotes individualized care based on people’s lives. They could improve the exchange of knowledge across the multidisciplinary team and reduce the provision of contradictory information, a known barrier (44). The Protocols were primarily designed for the context of multidisciplinary primary healthcare teams. However, they may also be tested in other settings, as they allow the professional (whether a nutritionist or not) to include recommendations for more specific nutritional needs while following the flowchart.

In **Box 2**, we present the continuation of the clinical case with a dietary advice approach conducted by a family physician using the Protocols.

**Figure:**
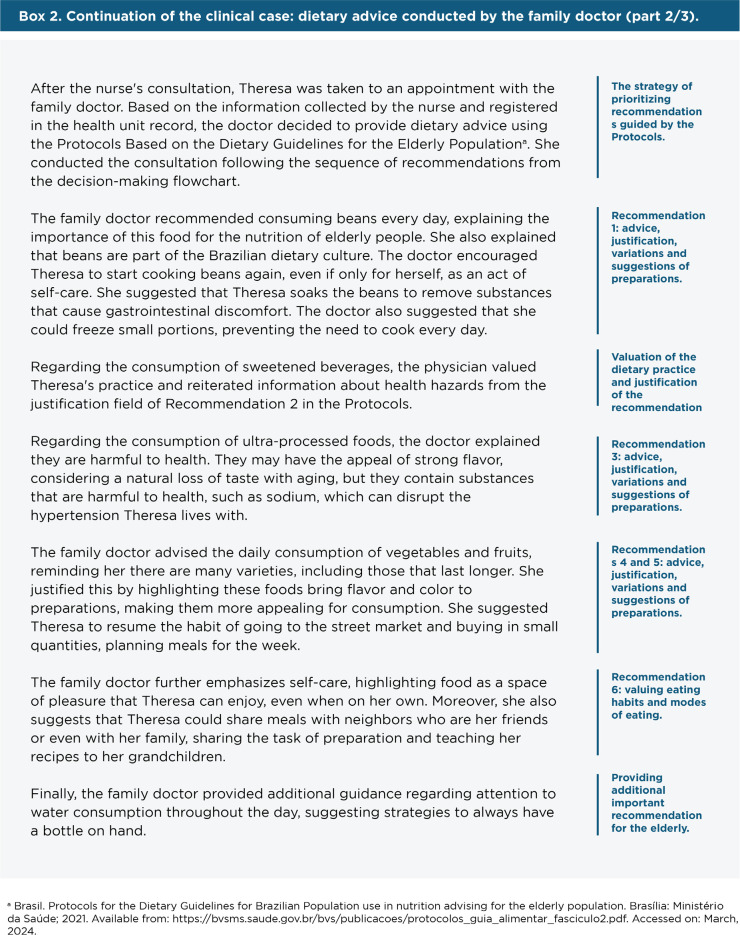

### Brazilian Dietary Guidelines-based Meal Plans

A meal plan is a personalized dietary prescription comprising structured daily menus, ideally crafted by a nutritionist. It delineates a meticulous inventory of foods and culinary preparations, along with recommended quantities. Typically, these plans are complemented by a substitute list, detailing “equivalent” foods within the same groups (grains, meats, vegetables, etc.), fostering diversity. Using meal plans proves advantageous in scenarios where individuals present clinical conditions requiring more intensive nutritional care, and where the nutritionist or the multi-professional team deems it necessary and feasible for the individual to adhere to a more systematic strategy to enhance diet quality (45).

Conventional meal plans used to focus primarily on achieving nutritional goals (46,47). In the past, this paradigm was considered adequate and sufficient, but advances in nutritional science have highlighted the need to incorporate other strategies and consider additional dietary characteristics that influence health. While meeting nutrient goals may be desirable, an approach with this sole objective is now considered inadequate (36).

Firstly, this approach reduces food to a reservoir of nutrients, disregarding that the physiological effects of these nutrients differ based on the characteristics of their food source (48-51). Their effects depend, for instance, on the degree of integrity of the food matrix, its cellular location, and interactions with other natural phytochemicals present in foods (50,52). Secondly, this approach neglects the effects of adding industrially modified substances to foods, such as food additives and modified proteins, carbohydrates, and fats. Lastly, this approach also overlooks the physiological effects of these nutrients may vary based on how different foods are combined, prepared, and consumed as meals (48,53). Considering that food processing is well known to impact food composition, the degree of food matrix integrity, and the patterns of food combination, Fardet (2024) argues that foods should first be chosen based on their list of ingredients (a proxy for processing), and then, if necessary, on the presence or absence of added salt, fat, and/or sugars, and not the other way around (54). Otherwise, diets can meet nutritional recommendations but still be unhealthy. Ebner and cols. (2022) described that 60% of industrialized foods scoring A/B with the Nutri-score labeling system (i.e. considered “good” products based on their nutrient profile) in France are ultra-processed, containing, for instance, plant protein isolates, taste enhancers, or artificial sweeteners (55).

Some nutritionists have certainly advanced in their clinical practices by adopting approaches that go beyond the exclusive focus on nutrients. In this paper, we aim to provide a guide for professionals to adopt these approaches in a more systematic way by presenting the proposal of Dietary Guidelines-based Meal Plans, a strategy that does not place the primary focus on nutrient profiles but rather on the overall eating pattern based on food processing characteristics. In this proposal, we use the dietary recommendations from the Brazilian Guidelines as the absolute primary theoretical framework. This proposal is based on two assumptions: 1) food processing is very well known to impact diet quality and the adherence to the dietary recommendations of the Brazilian Guidelines determines the energy intake, the nutritional profile, and various other important characteristics of the diet (described above in this paper), and 2) The nutrient profile can be assessed, if desired, in a second stage, where adjustments in the distribution of groups of natural or minimally processed foods within the meal plan can be made based on specific nutrient needs.

Therefore, this proposal places the selection of a diversity of foods and culinary preparations based on natural or minimally processed foods, focusing on tasteful, accessible and culturally appropriate choices, as the initial step. As a document providing general recommendations for the population, however, the Guidelines intentionally do not specify absolute quantities (portions, grams) recommended for consumption. Nevertheless, the document includes numerous recommendations that should guide the choice of foods/culinary preparations and the organization of meals within the meal plans. These recommendations draw extensive inspiration from the dietary practices of the segment of Brazilians who predominantly consume natural or minimally processed foods, identified in the 2008-2009 Food Intake Survey (56,57).

The recommendations of Guidelines that drive the development of the meal plans include: prioritizing the three main meals of the day - breakfast, lunch, and dinner - without substituting them for snacks; emphasizing the consumption of rice and beans, which is present in almost every major meal, although beans may occasionally be replaced for another legume; inclusion of vegetables in all lunches and dinners, prepared in various forms; restriction of red meats (beef or pork) to one-third of meals, with a preference for lean cuts and grilled or baked preparations, and the inclusion of other types of meats, eggs and vegetable-based dishes substituting them; incorporation of fruits, preferably consumed whole rather than as juice, into breakfast, as desserts for lunch and dinner, and occasionally as small snacks; and moderation in the use of oils, sugar, and salt, and abundance in the use of natural seasonings.

In the next step, these plans can be customized to individual energy requirements involving the establishment of desired quantities. The amounts of the selected foods and culinary preparations can be determined based on estimated energy requirements provided by predictive formulas that take into account information such as weight, height, and physical activity level. In addition, adjustments can be made based on strategic nutrient needs, defined based on individual characteristics such as life stage and clinical condition. The nutrient profile of the proposed plan can be compared with the recommended values of selected macronutrients and micronutrients (World Health Organization, the Institute of Medicine, or similar) and inform whether adjustments are necessary (58-65). The selection of prioritized micronutrients depends on the individual characteristics of each person, such as life stage, and clinical conditions.

Step-by-step to create a Brazilian Dietary Guidelines-based Meal Plan:

1. Assess individuals’ food consumption based on the Food Consumption Markers Questionnaire (31).
2. Structure meals (number, approximate times).
3. Select foods/culinary preparations based on the Dietary Guidelines (and organizing them into meals).
4. Estimate the total energy desired for the meal plan based on the calculation of estimated energy requirements for each person.
5. Propose quantities for the chosen foods/culinary preparations in Step 3 based on the estimates of energy established in Step 4. This requires using food composition tables.
6. Translate proposed quantities into household measurements (spoon, cup, etc.) based on reference tables. This will enable the individual to apply the plan in their dietary routine.
7. Compare the nutrient profile of the proposed plan with the recommendations of targeted macro and micronutrients, if desired.
8. If needed, make adjustments to foods/culinary preparations and their quantities.
9. Create a list of substitutes with equivalent portions for natural or minimally processed food groups (cereals, legumes, fruits, vegetables, meats, etc.).

In **Box 3**, we present the continuation of the clinical case with the dietary advice conducted by the nutritionist based on the development of a Brazilian Dietary Guidelines-based Meal Plan.

**Figure:**
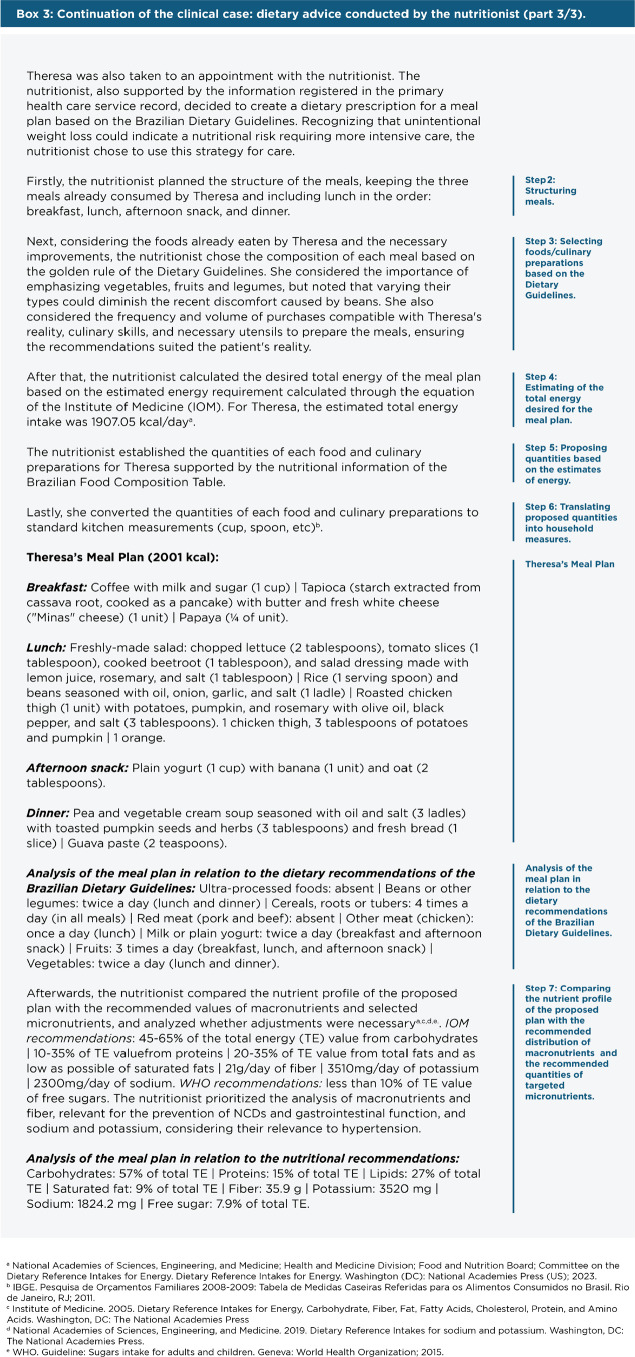

## DISCUSSION

Our paper describes two innovative strategies to translate the Brazilian Dietary Guidelines into clinical practice: the Protocols for individual dietary advice and the Dietary Guidelines-based Meal Plans. Given the importance of integrating nutrition actions into health systems and the potential of evidence-based guidelines for health promotion and NCD prevention, discussing how to effectively implement these strategies into daily appointments is highly relevant.

This is supported by evidence that providing individual dietary advice can be an effective strategy in promoting healthy eating, even in the face of some personal and structural barriers (5,7,30). The capacity of individuals to modify behaviors and develop self-care strategies, when properly supported, should not be underestimated. In clinical care, it is crucial for health professionals to recognize these barriers and offer support to overcome them, avoiding a paternalistic stance. The strategies presented in this paper, based on the Brazilian Dietary Guidelines, have the potential to empower individuals to make informed choices. While policies supporting structural changes are indispensable, the potential of competent educational and clinical interventions cannot be overlooked.

In the clinical case presented, the assessment of Theresa’s dietary practices based on the Food Consumption Markers Questionnaire provided strategic information to support healthcare professionals. The additional questions based on Theresa’s questionnaire answers enabled noting that her unhealthy habits were much related to a sense of loneliness. The family doctor used the Protocol for elderly people and was able to choose priority recommendations that could improve Theresa’s eating habits. The doctor focused on encouraging self-care and strategically advised her to resume consuming beans, crucial to ensure nutritionally balanced, flavorful, culturally appropriate meals, and to avoid ultra-processed foods, especially considering that she is a person with hypertension. The nutritionist, considering the severity of Theresa’s weight loss over the past year, decided to develop a meal plan based on the golden rule of the Dietary Guidelines, ensuring the resumption of lunch with the inclusion of beans, and adding another legume to dinner. Knowing that Theresa has been responsible for household chores and has culinary skills, the nutritionist ensured a plan with a variety of culinary preparations. Additionally, the nutritionist selected the micronutrients potassium and sodium for analyses, considering their role in the aetiology of hypertension, both of which had their recommended values met.

Our paper may support other countries to undertake similar approaches based on their own dietary guidelines. Uruguay, Ecuador, Mexico and Canada (66-70) are some of the countries that also consider the importance of food processing in their guidelines. Considering their local food culture, the nutritional conditions of their populations, and the organization of their healthcare services, these countries could develop similar approaches for their implementation in clinical practice. The government of Canada provides some additional resources for health professionals to apply Canada’s Dietary Guidelines, but none of them is a clinical protocol.

The implementation of the strategies proposed in this paper requires overcoming barriers among healthcare professionals related to both the technique of providing dietary advice and the lack of updated knowledge regarding theoretical frameworks of healthy eating. An Australian study assessed clinicians’ perspectives on barriers to implementing Mediterranean diet counselling into routine chronic disease care. Some of the points identified include limited knowledge and skills, variable understanding and acceptance of the scientific evidence, a legacy of single nutrient-based dietary education, and a lack of organizational culture for a dietary approach by the multidisciplinary team (71).

In Brazil, a randomized clinical trial tested the impact of 16 hours of face-to-face training based on the Dietary Guidelines for interprofessional teams working in primary care. The results showed that, compared to the control group, the intervention group increased knowledge, and self-efficacy, and incorporated more activities into their routine, related to the Dietary Guidelines (11,72). More recently, a project conducted in collaboration with the Ministry of Health, called QualiGuia, has developed a strategy to implement the Protocols into Primary Health Care considering continuing health education^1^. QualiGuia developed a massive open online course about the Protocols to allow widespread accessibility throughout the country. This course aims to support professionals to feel confident to incorporate these tools as a practical guide for dietary advice, however, the evaluation of its impact is still ongoing. Future studies should test the effectiveness of implementing the proposed strategies on process and impact indicators, considering the aim of improving the quality of healthcare professionals’ services and the population’s dietary habits.

In conclusion, our paper described a proposal to translate the Brazilian Guidelines into effective strategies that support professionals in clinical practice, potentially contributing to promoting healthier eating and reducing disease burden among the Brazilian population.

## Footnotes

1 The developed course is available in Portuguese on the SUS Open University platform, accessible at: https://www.unasus.gov.br/cursos/oferta/420030.
